# CELPI: trial protocol for a randomised controlled trial of a Carer End of Life Planning Intervention in people dying with dementia

**DOI:** 10.1186/s12877-022-03534-1

**Published:** 2022-11-16

**Authors:** G Arendts, L Chenoweth, BJ Hayes, E Campbell, M Agar, C Etherton-Beer, K Spilsbury, K Howard, G Braitberg, M Cubitt, C Sheehan, L Magann, T Sudharshan, LM Schnitker, J Pearce, I Gilmore, N Cerra, J duPreez, R Jaworski, S-C Soh, A Celenza

**Affiliations:** 1 Discipline of Emergency Medicine, Medical School, University of Western Australia, Perth, WA Australia; 2 Centre for Healthy Brain Ageing, School of Psychiatry, Faculty of Medicine, University of New South Wales, Sydney, Australia; 3 Northern Health Epping, Victoria, Australia; 4 Geriatric Medicine, Sir Charles Gairdner Hospital, Nedlands, WA Australia; 5 Centre for Improving Palliative, Aged and Chronic Care through Clinical Research and Translation, University of Technology, Sydney, Australia; 6 WA Centre for Health and Ageing, University of Western Australia, Perth, WA Australia; 7 University of Notre Dame, Fremantle, WA Australia; 8 School of Public Health, University of Sydney, Camperdown, NSW Australia; 9 Emergency Medicine, Austin Health, Victoria, Australia; 10 Emergency Medicine, The Royal Melbourne Hospital, Victoria, Australia; 11 Palliative Care Medicine, St George Hospital, NSW Kogarah, Australia; 12 Emergency Medicine, Liverpool Hospital, Sydney, Australia; 13 School of Nursing, Queensland University of Technology, Brisbane, Australia; 14 Fiona Stanley Hospital, Murdoch, WA Australia

**Keywords:** Dementia, Palliative care, Person-centred care, Randomised controlled trial, Emergency medicine

## Abstract

**Background:**

Dementia is a leading cause of death in developed nations. Despite an often distressing and symptom laden end of life, there are systematic barriers to accessing palliative care in older people dying of dementia. Evidence exists that 70% of people living with severe dementia attend an emergency department (ED) in their last year of life. The aim of this trial is to test whether a Carer End of Life Planning Intervention (CELPI), co-designed by consumers, clinicians and content specialists, improves access to end of life care for older people with severe dementia, using an ED visit as a catalyst for recognising unmet needs and specialist palliative care referral where indicated.

**Methods:**

A randomised controlled trial (RCT) enrolling at six EDs across three states in Australia will be conducted, enrolling four hundred and forty dyads comprising a person with severe dementia aged ≥ 65 years, and their primary carer.

Participants will be randomly allocated to CELPI or the control group. CELPI incorporates a structured carer needs assessment and referral to specialist palliative care services where indicated by patient symptom burden and needs assessment. The primary outcome measure is death of the person with dementia in the carer-nominated preferred location. Secondary outcomes include carer reported quality of life of the person dying of dementia, hospital bed day occupancy in the last 12 months of life, and carer stress. An economic evaluation from the perspective of a health funder will be conducted.

**Discussion:**

CELPI seeks to support carers and provide optimal end of life care for the person dying of dementia. This trial will provide high level evidence as to the clinical and cost effectiveness of this intervention.

**Trial registration:**

ACTRN12622000611729 registered 22/04/2022.

**Supplementary Information:**

The online version contains supplementary material available at 10.1186/s12877-022-03534-1.

## Background

One in every four people who reach the age of 65 will die of or with dementia, yet death from dementia is “under-recognised and stigmatised” [1]. In people with advanced dementia, the final year of life is characterised by a trajectory of progressive severe disability and health problems such as pain, pressure injury, infections, eating difficulties and changed behaviours [2, 3]. The physical symptoms are not dissimilar to those experienced by people with terminal cancer. However, compared to the relatively well established link between terminal cancer and specialist palliation, referral pathways to access palliative care for the population dying of advanced dementia, and what that palliative care looks like, are disorganised and difficult to qualify [4].

Barriers to the uptake of palliative care include lack of understanding of the disease trajectory of advanced dementia (with only one third of family carers understanding that severe dementia is a terminal illness) [5]; a lack of responsibility for initiation of the process; and misapprehension that end of life planning is synonymous with withholding essential treatment [6]. Palliative care in people with advanced dementia is further undermined by i) a lack of fully validated symptom scales that can identify the range of cognitive, physiological, emotional and behavioural symptoms occurring in late-stage dementia; ii) incomplete information available about the healthcare and lifestyle preferences and values of a person who lacks capacity to communicate these; and iii) the frequent involvement of different medical specialties, who may focus on individual diseases or organ systems and not recognise that the person is approaching the end of their life, or is now dying.

Over 70% of people dying of and with dementia attend an emergency department (ED) at least once in their last 12 months of life, with the highest rates in those with other comorbidities and living with a carer at home [7]. This raises the opportunity of using the ED visit as a "teachable moment", helping bridge the gap between the sometimes-abstract aspects of care planning and the realities of acute illness. Using the ED visit as the launching pad to initiate an intervention that will improve end of life care for people with severe dementia would invert the usual negative experiences of an ED, where the expectations of dementia-friendly care are almost never met [8].

The objective of this randomised controlled trial (RCT) is to investigate the impact of a Carer End of Life Planning Intervention (CELPI) to determine whether this results in a meaningful improvement in outcomes for people with severe dementia approaching end of life who attend an ED, and their carers.

## Methods

### Design

**Fig. 1:**
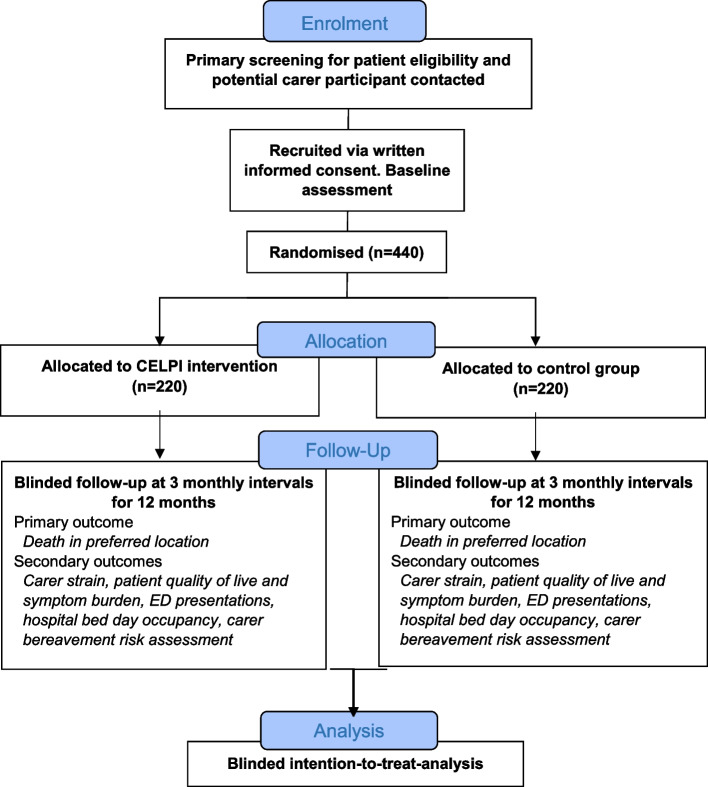
Participant flow

A single-blind multi-centre parallel RCT of CELPI compared to usual post-discharge care will be conducted (Fig. 1).

### Participants and setting

We will enrol dyads of a person with dementia and their carer, with the carer as determined by relevant state legislation. People with dementia aged at least 65 years old attending one of six enrolling EDs will be screened after attendance for trial inclusion and exclusion criteria. The EDs are all metropolitan tertiary referral EDs located in Melbourne, Perth and Sydney, Australia.

#### Inclusion Criteria

The person with dementia must have ALL of the following:

1. Established prior documented diagnosis of dementia, supported by an appropriate assessment screen e.g. mini mental state exam (MMSE) [9]. or Montreal Cognitive Assessment (MoCA) < 13/30
2. Functional Assessment Staging Test (FAST) Stage 6d-7f [10].
3. A carer residing within a 30 km travel distance of the enrolling ED or, if beyond that radius, agreeable to using telehealth to undertake the intervention.
4. Medicare eligible

##### Exclusion Criteria:

1. No identifiable singular adult carer as a surrogate medical decision maker
2. Person with dementia is under the care of the Public Guardian
3. Clinician judgement that death is imminent (within one week)
4. Person with dementia is a current patient of a specialist palliative care service, or a prior patient within the preceding 12 months.
5. Significant symptoms (e.g. pain, dyspnoea) requiring a referral to specialist palliative care on this presentation.
6. Carer requires translation of written and spoken language

People with dementia will be eligible to participate in this RCT if they reside in residential aged care or the community, and if they are discharged home from the ED or admitted to an inpatient ward.

### Sample size

The study is powered to detect a significant difference in the primary outcome of death within the follow up period in the carer supported preferred location. Where the person with dementia has previously indicated a preferred location, this will be ascertained from and confirmed with the carer. Otherwise, the carer will be requested to nominate. Whilst there are large datasets that describe the location of death of people with advanced dementia, only two small studies from a recent systematic review provided data on preferred location of death [11].

Using this review to assume 60% of people with dementia in the control arm of the trial will die in a carer preferred location, then 408 participants will need to be enrolled in the study to have 90% power to detect a 15% increase in the proportion of people dying in preferred locations to 75%. The Type I error probability associated with these tests of this null hypothesis is 0.05. Applying a small (< 10% tolerance) to incomplete data and loss to follow up, we will enrol 440 participants, 220 per study arm.

### Participant screening and recruitment

There will be a multi-pronged approach to screening and recruitment. A research assistant (the recruiter) working in each of the six EDs will screen electronic records on a daily basis to identify potential people with dementia based on age and available triage information. This will be augmented by direct referrals of potentially eligible patients by hospital clinicians (medical, nursing and allied health) working in the ED. Based on available information, in person or telephone contact will be made with the identified primary carer, where verbal permission will be obtained to continue the screening for inclusion and exclusion criteria with the carer and patient. Once it is determined the patient-carer dyad is eligible for the trial, the carer will be provided with a paper or electronic scanned version (based on their preference) of the trial information and consent form. Signed consent forms will be returned to the recruiter by mail or electronically.

### Baseline assessment

The baseline assessment will be undertaken by the recruiter within seven days of receipt of a signed consent form. This will usually be via telephone with the carer, and will comprise where applicable measures that have undergone prior psychometric validation and are fit for purpose:

1. Carer-informed patient baseline assessment
  - Past medical history and demographic details, hospitalisations in the prior twelve months. Co-morbidity will be codified using the Charlson Comorbidity Index [12]. Carer information will be confirmed with hospital and other written medical records where available.
  - Australia-modified Karnofsky Performance Status (AKPS) [13].
  - Quality of Life in Late Stage Dementia (QUALID)[14].
  - Symptoms using the Symptom Assessment Scale (SAS) [15]. Although designed to be used by patients to self-report, it has been used for proxy reporting.
  - Preferred location of death. We will capture what was known (if anything) of the preferred location of the person with dementia, but use the carer nominated location for the purpose of the primary outcome adjudication.
2. Carer baseline assessment
  - Demographic details
  - Quality of life (EQ5D) [16].
  - Modified Caregiver Strain Index (MCSI) [17].

### Randomisation and control arm

After baseline assessment by the recruiter, an intervention (CELPI) clinician will be notified by email and will randomly assign participants into one of the two study arms using a web-based randomisation service. This will ensure the recruiter, who will also conduct the three monthly follow up assessments, is blinded to the allocation arm. Randomisation will be in a 1:1 ratio stratified by city of enrolment.

Once randomised, the CELPI clinician, who will have a tertiary degree in a health discipline (e.g. psychology, occupational therapy) but not be a physician, will contact all participants regardless of allocation arm and provide education regarding publicly available resources for the carers of people with dementia. The clinician will have all details of the baseline assessment available to them, such that they will have sufficient information on the person with dementia and their carer, and not need to access hospital records. If in the control arm, the clinician will then have no further contact with the participant. Participants in the control group will receive usual care from all health professionals who are involved in their management during the 12-month follow up. No treatments will be withheld from the control group.

### Intervention arm

The basis of the CELPI intervention is a holistic needs assessment followed by an agreed referral plan to address needs identified by that assessment. Participants will receive at least one visit from the CELPI clinician that will comprise a structured needs assessment using the CANDID tool [18] developed for this trial (Appendix 1). CANDID is designed to capture information on the current and future needs of both the carer and the person with dementia, as well as clarifying what, if any, advance care planning has been done to date. The assessment will be used to develop and implement tailored follow up actions to address these needs mapped to existing available services, but focussing on the two key components of the intervention.

i what a palliative care referral would entail; and.
ii what a palliative approach and advance care planning looks like for a person dying with dementia, stressing at all times that the goal is to align care with the wishes of the person with dementia regarding how they would want their end of life to be. CANDID will capture any prior advance health directive or care plan made by the person with dementia when they had capacity, as well as any prior goals of care discussions already held with the carer.

CELPI will therefore focus on identifying current needs; likely future complications; and if necessary, guiding carer-initiated treatments for distressing symptoms.

CELPI clinicians (one per state) will be currently registered health professionals with experience in the care of people with cognitive impairment and/or palliative care needs, who will receive structured training as members of the research team. This will include education about advanced dementia, the use of CANDID and techniques such as motivational interviewing and goal setting [19]. The competencies and skills of the clinicians will be assessed to ensure fidelity across clinicians and over time through a fidelity checklist developed for the trial (Appendix 2).

The role of the CELPI clinician is not to replace the role of usual treating health care professionals or to provide new services. Instead, the clinician will act as a navigator to help carers understand the CANDID findings, make guided decisions about palliative care and care planning, and develop an action plan for referring and linking the carer into specialist palliative care or dementia services as indicated. This will be supported by the provision of educational materials and a written summary provided to the carer and the GP of the person with dementia. Broadly, reasons for referral to a specialist palliative care service will fall into at least one of three groups: physical symptoms; complex social situations; and anticipated complex grief.

### Outcome measures

The primary outcome measure is the proportion of participants dying in their carer-nominated preferred location for death within 12 months of enrolment. The person-centred secondary outcome measures are changes in carer (proxy) reported and self-ratings of quality of life, symptoms and strain, using the same measurements done at baseline. Other secondary outcome measures will be health system focussed, namely.

1. Number of ED attendances post enrolment (with and without subsequent admission to hospital)
2. Hospital occupied bed days post enrolment, including hospital in the home admissions
3. Days spent in nominated preferred location of care post enrolment
4. Number and type of medical interventions in last seven days of life e.g. IV fluids or antibiotics

Finally, if the person with dementia dies in the follow up period, we will measure bereavement risk in the carer using the Modified Bereavement Risk Index (MBRI) [20].

A recruiter blinded to the allocation arm of the carer will undertake all outcome measure assessments every three months post enrolment, for a maximum of twelve months. Participants will have the choice to enter questionnaire responses directly into an electronic database, or answer questions by phone with the recruiter recording their responses for them.

### Statistical Analysis

Publication of a full statistical analysis plan separate to this manuscript is planned. This will include details on planned interim and subgroup analyses. Outcome analyses will be undertaken on an intention-to-treat basis by the study statistician blinded to group allocation. The overall difference in proportion of participants dying in a preferred location between the intervention and control groups will be assessed using logistic regression. Secondary outcome analyses of quality of life and strain measures, collected multiple times from the same patient-carer dyad, will be performed by constructing random effects models with the intervention group variable interacted with time to account for within-dyad correlation. Health system outcomes will be assessed by including the intervention group variable in an appropriate count-based regression model that accounts for the variable exposure (length of time before death). Inclusion of baseline data in regression models will be used to explore additional variation within dyad characteristics.

Elements introduced to mitigate bias in the study include use of a computer randomisation service, blinded outcome assessment and intention to treat analysis.

### Economic Evaluation Plan

A trial-based economic evaluation will take the perspective of the health care funder. Health care and intervention costs will be collected over the trial period. We will collect data on the cost to deliver the intervention (including staff costs, training, capital costs and consumables) as well as human and other resource costs associated with inpatient hospital admissions, ED presentations and other health service contacts. Inpatient admissions will be costed based upon Australian Refined Diagnosis Related Groups costs from the National Hospital Cost Data Collection; ED presentations will be costed using data from Independent Hospital Pricing Authority. All costs and savings will be adjusted to the base year using published deflators, and, if needed, costs incurred beyond one year will be discounted using a standard 5% discount rate.

Using mean costs and mean health outcomes in each trial arm, the incremental costs per 1) hospital death avoided; and 2) hospitalisation avoided compared with control group will be calculated; results will be plotted on a cost-effectiveness plane. Bootstrapping will be used to estimate a distribution around costs and health outcomes, and to calculate the confidence intervals around the incremental cost-effectiveness ratios. One-way and multi-way sensitivity analyses will be conducted around key variables and a probabilistic sensitivity analysis will estimate joint uncertainty in all parameters. A cost-effectiveness acceptability curve will be plotted to provide information about the probability that the intervention is cost-effective, given willingness to pay for each unit of benefit gained.

### Patient and Public Involvement

Authors IG and NC are consumer representatives with lived experience as carers of people that died of dementia and attended ED. They have contributed to the design of the recruitment strategy, intervention, the active control arm of the trial and the outcome measures.

## Discussion

There is very little high quality trial evidence to guide intervention in people dying of dementia. The most recent Cochrane review from 2016 found only one trial randomised at the individual participant level (*n* = 99) and concluded “that there is insufficient evidence to assess the effect of palliative care interventions in advanced dementia” [21]. A recent pilot RCT from the USA (*n* = 62) showed the feasibility and possible benefit of palliative intervention in this population [22].

Landmark research has shown that providing prognostic certainty amongst carers of people with advanced dementia substantially alters the rate of futile and invasive interventions before death [23]. Prior work shows ED attendance follows a predictable course in the last year of life, AND that course is highly modifiable through the use of palliative care [7].

This RCT will test an intervention co-designed by consumers with lived experience as carers of people who died of dementia, clinicians, and content specialists. It builds on prior trial learnings, notably a pilot of a trial led by some members of our team in Western Australia. CELPI will help carers make guided decisions to navigate the healthcare system to access specialist palliative care and other dementia services. CELPI will be delivered by a trained clinician and will incorporate education and coordination.

The intervention in this trial is focussed at the level of the carer. As noted in a recent position paper “proxy decision making can be confounded as such decisions may be impossible to separate from the family carers’ own views and furthermore, where the family carer has supportive (or other) care needs of their own” [24]. With this in mind, we have designed the CANDID measure to assess not only the needs of the person with dementia, but the needs of the carer, and will employ a suite of secondary outcome measures to assess carer strain and quality of life. The primary outcome of this trial (place of death) is commonly used in palliative care trials, however we recognise in many instances the person with dementia with capacity may not have had conversations with the carer, or provided written directives as to their preferred location of death.

A crucial feature in the design of this RCT is an active control arm, with all participants after randomisation receiving educational resources. While this study design element may risk contamination and dilution of the effect of the intervention, it was deemed crucial by our consumer investigators to provide all participants with the opportunity to seek help for a person who has been identified in the trial screening as approaching end of life with dementia. We believe the additional elements of the intervention are sufficient to trial whether this improves outcome over and above the passive provision of educational resources.

The research outcomes have potential to change current end of life practice and policies for older people dying of dementia and presenting to an ED. The findings from this project could positively impact on the design and implementation of end of life programs in Australia and internationally for people dying of dementia. A publication plan for the trial will be through peer review journals adjudicating the clinical and economic endpoints, with the latter particularly crucial for demonstrating the future benefits of investing in palliative care for people dying of dementia. Health policy changes arising from this trial will be advanced via the extensive clinical networks and health department links of the research team. We intend to launch any successful research report in conjunction with carer support organisations, advocating for policy changes to support carers.

## Supplementary Information

**Additional file 1.** Carer Needs Assessment.

**Additional file 2.** Fidelity Checklist.

## Data Availability

The datasets used and/or analysed during the current study are available from the corresponding author on reasonable request.

## Abbreviations

ED: Emergency Department
CELPI: Carer End of Life Planning Intervention
RCT: Randomised Controlled Trial
MMSE: Mini Mental State Examination
MoCA: Montreal Cognitive Assessment
FAST: Functional Assessment Staging Test
AKPS: Australia-modified Karnofsky Performance Status
QUALID: QUAlity of Life in late stage Dementia
SAS: Symptom Assessment Scale
MCSI: Modified Carer Strain Index
MBRI: Modified Bereavement Risk Index
RGS: Research Governance Service
ACTRN: Australian Clinical Trials Registration Number

## References

[1] Harrison KL, Hunt LJ, Ritchie CS (2019). Dying With Dementia: Underrecognized and Stigmatized. J Am Geriatr Soc.

[2] Sampson EL, Burns A, Richards M (2011). Improving end-of-life care for people with dementia. Br J Psych.

[3] Mitchell SL, Teno JM, Kiely DK (2009). The clinical course of advanced dementia. N Engl J Med.

[4] Stewart JT, Schultz SK (2018). Palliative Care for Dementia. Psychiatr Clin North Am.

[5] Gabbard J, Johnson D, Russell G (2020). Prognostic Awareness, Disease and Palliative Understanding Among Caregivers of Patients With Dementia. Am J Hosp Palliat Care.

[6] Russ A, Mountain D, Rogers IR (2015). Staff perceptions of palliative care in a public Australian, metropolitan emergency department. Emerg Med Australas.

[7] Rosenwax L, Spilsbury K, Arendts G (2015). Community-based palliative care is associated with reduced emergency department use by people with dementia in their last year of life: A retrospective cohort study. Palliat Med.

[8] Schnitker LM, Martin-Khan M, Burkett E (2015). Process quality indicators targeting cognitive impairment to support quality of care for older people with cognitive impairment in emergency departments. Acad Emerg Med.

[9] Tombaugh TN, McIntyre NJ (1992). The mini-mental state examination: a comprehensive review. J Am Geriatr Soc.

[10] Sclan SG, Reisberg B. Functional assessment staging (FAST) in Alzheimer's disease: reliability, validity, and ordinality. Int Psychogeriatr. 1992;4 Suppl 1:55–69. 10.1017/s1041610292001157. 10.1017/s1041610292001157 1504288

[11] Badrakalimuthu V, Barclay S (2014). Do people with dementia die at their preferred location of death? A systematic literature review and narrative synthesis. Age Ageing.

[12] Charlson ME, Pompei P, Ales KL (1987). A new method of classifying prognostic comorbidity in longitudinal studies: development and validation.

[13] Abernethy AP, Shelby-James T, Fazekas BS, et al. The Australia-modified Karnofsky Performance Status (AKPS) scale: a revised scale for contemporary palliative care clinical practice [ISRCTN81117481]. BMC Palliat Care. 2005;4:7. 10.1186/1472-684X-4-7. 10.1186/1472-684X-4-7 PMC1308820 16283937

[14] Sopina E, Chenoweth L, Luckett T (2019). Health-related quality of life in people with advanced dementia: a comparison of EQ-5D-5L and QUALID instruments. Qual Life Res.

[15] Daveson BA, Allingham SF, Clapham S (2021). The PCOC Symptom Assessment Scale (SAS): A valid measure for daily use at point of care and in palliative care programs. PLoS ONE [Electronic Resource].

[16] Luo N, Johnson JA, Shaw JW (2005). Self-reported health status of the general adult U.S. population as assessed by the EQ-5D and Health Utilities Index. Med Care.

[17] Jennings LA, Reuben DB, Evertson LC (2015). Unmet needs of caregivers of individuals referred to a dementia care program. J Am Geriatr Soc.

[18] O'Connor M, Campbell E, du Preez J, et al. Time to Talk CANDIDly about Dementia. Oceanic Palliative Care Conference. 2021.

[19] Ko E, Hohman M, Lee J (2016). Feasibility and Acceptability of a Brief Motivational Stage-Tailored Intervention to Advance Care Planning: A Pilot Study. Am J Hosp Palliat Care.

[20] Sealey M, Breen LJ, O'Connor M, Aoun SM. A scoping review of bereavement risk assessment measures: Implications for palliative care. Palliat Med. 2015;29(7):577–89. 10.1177/0269216315576262. Epub 2015 Mar 24. 10.1177/0269216315576262 25805738

[21] Murphy E, Froggatt K, Connolly S (2016). Palliative care interventions in advanced dementia. Cochrane Database Syst Rev.

[22] Hanson LC, Kistler CE, Lavin K (2019). Triggered Palliative Care for Late-Stage Dementia: A Pilot Randomized Trial. J Pain Symptom Manage.

[23] Loizeau AJ, Shaffer ML, Habtemariam DA (2018). Association of Prognostic Estimates With Burdensome Interventions in Nursing Home Residents With Advanced Dementia. JAMA Intern Med.

[24] Fox S, FitzGerald C, Harrison Dening K (2017). Better palliative care for people with a dementia: summary of interdisciplinary workshop highlighting current gaps and recommendations for future research. BMC Palliat Care.

