# Supplementary material for: CELPI: trial protocol for a randomised controlled trial of a Carer End of Life Planning Intervention in people dying with dementia
Source: BMC Geriatr. 2022 Nov 16;22:869. doi: 10.1186/s12877-022-03534-1 (PMC9670369; doi:10.1186/s12877-022-03534-1)
Supplement: Supplementary file 1 — Additional file 1. Carer Needs Assessment. [file 12877_2022_3534_MOESM1_ESM.docx]

**Appendix 1 – Needs Assessment**

**CARER END OF LIFE PLANNING INTERVENTION**

**CARER- DIRECTED NEEDS ASSESSMENT**

| NAME OF CARER | |  | | DOB |  | M | F | OTHER |
| --- | --- | --- | --- | --- | --- | --- | --- | --- |
| NAME OF CARE RECIPIENT | |  | | | | | | |
| RELATIONSHIP TO CARE RECIPIENT | |  | | | | | | |
| IN-HOME CARE ADDRESS | | | RESIDENTIAL AGED CARE FACILITY ADDRESS | | | | | |
|  | | |  | | | | | |
|  | | |  | | | | | |
|  | | |  | | | | | |
|  | | |  | | | | | |
| **EMAIL ADDRESS:** |  | | | | | | | |
| NAME OF INTERVENTION CLINICIAN | |  | | | | | | |
| DATE OF INTERVIEW | |  | | | | | | |
| DURATION OF INTERVIEW | |  | | | | | | |

This questionnaire has been developed to assist you in identifying your current and future care needs so that we can best support you. Together, we will determine what your care recipient’s and your needs are currently and what they may be in the future. Together you and I will design a care plan to ensure that you and your care recipient are supported throughout the dementia journey.

**CARERS PERCEPTION OF THEIR CARE RECIPIENT’S CURRENT NEEDS:**

- **Use this adapted/modified Symptom Assessment Scale (SAS) data to verify and establish current needs and anticipated/perceived future needs.**
- **Use opportunity to provide carer education on the use of the SAS**

| **Carer Rated Score** | **Symptom Assessment Scale (0-10)** *Rate experience of symptom distress over a 24hr period 0 = absent 10 = worst possible*  0 = Continue care 1 -3 = Monitor and record 4-7 = Review/change plan of care; referral, intervention as required 8-10 = Urgent action | | | | | | | | | | | | | | |
| --- | --- | --- | --- | --- | --- | --- | --- | --- | --- | --- | --- | --- | --- | --- | --- |
|  | Distress from difficulty sleeping |  |  |  |  |  |  |  |  |  |  |  |  |  |  |
|  | Distress from Appetite |  |  |  |  |  |  |  |  |  |  |  |  |  |  |
|  | Distress from Nausea |  |  |  |  |  |  |  |  |  |  |  |  |  |  |
|  | Distress from Bowels |  |  |  |  |  |  |  |  |  |  |  |  |  |  |
|  | Distress from Breathing |  |  |  |  |  |  |  |  |  |  |  |  |  |  |
|  | Distress from Fatigue |  |  |  |  |  |  |  |  |  |  |  |  |  |  |
|  | Distress from Pain |  |  |  |  |  |  |  |  |  |  |  |  |  |  |
|  | Other |  |  |  |  |  |  |  |  |  |  |  |  |  |  |
|  | **Rated by Carer** |  |  |  |  |  |  |  |  |  |  |  |  |  |  |

| **Problem Severity Score Actions (0-3)** *Refer to complete definition and rate each domain*  0 = Continue care 1 = Monitor and record 2 = Review/change plan of care; referral, intervention as required 3 = Urgent action | | | | | | | | | | | | | | |
| --- | --- | --- | --- | --- | --- | --- | --- | --- | --- | --- | --- | --- | --- | --- |
| Pain |  |  |  |  |  |  |  |  |  |  |  |  |  |  |
| Other Symptoms |  |  |  |  |  |  |  |  |  |  |  |  |  |  |
| Psychological / Spiritual |  |  |  |  |  |  |  |  |  |  |  |  |  |  |
| Family / Carer |  |  |  |  |  |  |  |  |  |  |  |  |  |  |

| **Palliative Care Phase**  **Stable =** Monitor **Unstable =** Urgent action required **Deteriorating =** Review plan of care **Terminal =** Provide EOL care |
| --- |

| **Problem Severity Score Actions (0-3)** *Refer to complete definition and rate each domain*  0 = Continue care 1 = Monitor and record 2 = Review/change plan of care; referral, intervention as required 3 = Urgent action | | | | | | | | | | | | | | |
| --- | --- | --- | --- | --- | --- | --- | --- | --- | --- | --- | --- | --- | --- | --- |
| Pain |  |  |  |  |  |  |  |  |  |  |  |  |  |  |
| Other Symptoms |  |  |  |  |  |  |  |  |  |  |  |  |  |  |
| Psychological / Spiritual |  |  |  |  |  |  |  |  |  |  |  |  |  |  |
| Family / Carer |  |  |  |  |  |  |  |  |  |  |  |  |  |  |

| **Palliative Care Phase**  **Stable =** Monitor **Unstable =** Urgent action required **Deteriorating =** Review plan of care **Terminal =** Provide EOL care |
| --- |

- Does the care recipient meet criteria for specialist palliative care referral?
- Does the carer want a referral?
- Explain the referral process for Silverchain or MPaCCS

**ADVANCE CARE PLANNING**

| **Has anyone had an end-of-life discussion with you or your care-recipient?** |  |
| --- | --- |
| **Has anyone discussed an Advance Care Plan with him/her?** |  |
| **Is there a documented Advance Care Plan?** |  |
| **Would you like to know more about an Advance Care Plan?** |  |
| **What do you think matters most to your care recipient right now?** |  |
| **What do you think he/she may need right now?** |  |
| **Where would your care recipient prefer to be cared for?** |  |
| **Where would he/she like to be when they pass away?** |  |
| **Are there any particular rituals he/she would like to see actioned as part of their death experience, after-death experience?** |  |

**CARERS PERCEPTION OF THEIR CARE RECIPIENT’S FUTURE NEEDS**

| **Is there anything you find difficult to talk to your care recipient about?** |  |
| --- | --- |
| **Is there anything you find difficult to talk to family members about?** |  |
| **What are your care recipient’s spiritual beliefs?** |  |
| **In your opinion, do they have any fears or concerns about death or dying?** |  |

**CARER’S CURRENT NEEDS**

| **What concerns you most about your care recipient right now?** |  |
| --- | --- |
| **Are you aware that they will die?** |  |
| **Are there any aspects of your care recipient’s disease that you do not understand or would like to know more about?** |  |
| **Do you understand the stages of dementia? Would you like to discuss this now?** |  |
| **Do you have any concerns related to your own health today?** |  |
| **How do you feel about taking time for yourself away from your caring role?** |  |
| **Do you have any financial concerns?** |  |
| **Are there any legal issues you need addressed?** |  |
| **What kind of support do you have from your family?** |  |
| **Do you have any practical help/ services in the home?** |  |
| **Are you satisfied with the level of service provision being provided?** |  |

**EQUIPMENT NEEDS:**

**Is there any equipment you feel you need to assist you in caring for your care recipient?**

| **Shower chair** |  |
| --- | --- |
| **Detachable shower hose** |  |
| **Non-slip mat** |  |
| **Hoist** |  |
| **Portable commode** |  |
| **Over-toilet frame** |  |
| **Hand rails** |  |
| **OTHER:** |  |
|  |  |

**SYMPTOM MANAGEMENT:**

**Is there any aspect of their care that you would like more information about?**

| **Pain management** |  |
| --- | --- |
| **Eating and drinking** |  |
| **Nutrition** |  |
| **Personal grooming** |  |
| **Bathing/showering** |  |
| **Avoiding pressure wounds** |  |
| **Behaviour management i.e. anxiety, aggression, apathy** |  |
| **Communication** |  |
| **Delirium** |  |
| **Falls prevention** |  |
| **Incontinence** |  |
| **Toileting** |  |
| **Shortness of breath** |  |
| **Fatigue** |  |
| **Decision making** |  |

**MIDDLE OF THE NIGHT EMERGENCY**

In an emergency, who would you call?

**Do you have contact numbers of various services that could provide you with help?**

| CONTACT’S NAME: | CONTACT NUMBER: |
| --- | --- |
| **General Practitioner most familiar with situation** |  |
| **Silverchain** |  |
| **Psychological Counsellor** |  |
| **Family member** |  |
| **Ambulance** |  |
| **Funeral director** |  |

**CARERS PERCEIVED FUTURE NEEDS:**

| **What concerns you the most about the future?** |  |
| --- | --- |
| **Do you have any concerns about your future caring role?** |  |
| **What are your spiritual beliefs?** |  |
| **What does that look like for you?** |  |
| **Are there any particular rituals you would like to see actioned as your care recipient reaches the end of their life?** |  |

**PROPOSED CURRENT CARE PLAN:**

| IDENTIFIED AREAS OF NEED: | ACTION TAKEN | REFERRAL MADE TO | DATE | REVIEW DATE |
| --- | --- | --- | --- | --- |
|  |  |  |  |  |
|  |  |  |  |  |
|  |  |  |  |  |
|  |  |  |  |  |
|  |  |  |  |  |
|  |  |  |  |  |
|  |  |  |  |  |

**PROPOSED FUTURE CARE PLAN:**

| IDENTIFIED POTENTIAL AREAS OF NEED: | ACTION | REFER TO | DATE | REVIEW  DATE |
| --- | --- | --- | --- | --- |
|  |  |  |  |  |
|  |  |  |  |  |
|  |  |  |  |  |
|  |  |  |  |  |
|  |  |  |  |  |
|  |  |  |  |  |
|  |  |  |  |  |

**ADDITIONAL NOTES:**
