# Supplementary material for: CELPI: trial protocol for a randomised controlled trial of a Carer End of Life Planning Intervention in people dying with dementia
Source: BMC Geriatr. 2022 Nov 16;22:869. doi: 10.1186/s12877-022-03534-1 (PMC9670369; doi:10.1186/s12877-022-03534-1)
Supplement: Supplementary file 2 — Additional file 2. Fidelity Checklist. [file 12877_2022_3534_MOESM2_ESM.docx]

**Appendix 2 - Checklist**

**FIDELITY**

**FACE-TO-FACE INTERVIEW**

| PART. ID: | FIDELITY CHECKLIST | DATE COMPLETED |
| --- | --- | --- |
|  | **Completed CELPI CARER DIRECTED NEEDS Ax** |  |
|  | **Educated carer on use of the Symptom Assessment Scale (SAS)** |  |
|  | **Using the SAS, identified carers perceptions about care recipient’s current symptoms.** |  |
|  | **Identified carer’s understanding of symptom management. Provided education where appropriate or flagged for referral.** |  |
|  | **Determined current needs for equipment** |  |
|  | **Confirmed carer’s understanding of stages of dementia and the dying process. Provided education if the carer was willing.** |  |
|  | **Confirmed whether an ACP has been actioned. If not, provided education and resources and flagged for referral** |  |
|  | **Determined carer’s perception of their care recipient’s future needs** |  |
|  | **Determined whether the care recipient met criteria for specialist palliative care referral** |  |
|  | **Explained the referral process for Silverchain or MPaCCS** |  |
|  | **Determined whether or not the carer wanted a referral?** |  |
|  | **Determined carer’s current needs** |  |
|  | **Determined carer’s perceived future needs** |  |
|  | **Documented proposed current care plan, actions to be taken and dates actioned** |  |
|  | **Documented proposed future care plan, actions to be taken and dates to be actioned** |  |
